# Age-Dependent and Sleep/Seizure-Induced Pathomechanisms of Autosomal Dominant Sleep-Related Hypermotor Epilepsy

**DOI:** 10.3390/ijms21218142

**Published:** 2020-10-30

**Authors:** Kouji Fukuyama, Motohiro Okada

**Affiliations:** Department of Neuropsychiatry, Division of Neuroscience, Graduate School of Medicine, Mie University, Tsu, Mie 514-8507, Japan; k-fukuyama@clin.medic.mie-u.ac.jp

**Keywords:** autosomal dominant sleep-related hypermotor epilepsy, L-glutamate, hemichannel, basal ganglia, extracellular signal-regulated kinase, protein kinase B

## Abstract

The loss-of-function S284L-mutant α4 subunit of the nicotinic acetylcholine receptor (nAChR) is considered to contribute to the pathomechanism of autosomal dominant sleep-related hypermotor epilepsy (ADSHE); however, the age-dependent and sleep-related pathomechanisms of ADSHE remain to be clarified. To explore the age-dependent and sleep-induced pathomechanism of ADSHE, the present study determined the glutamatergic transmission abnormalities associated with α4β2-nAChR and the astroglial hemichannel in the hyperdirect and corticostriatal pathways of ADSHE model transgenic rats (S286L-TG) bearing the rat S286L-mutant *Chrna4* gene corresponding to the human S284L-mutant *CHRNA4* gene of ADSHE, using multiprobe microdialysis and capillary immunoblotting analyses. This study could not detect glutamatergic transmission in the corticostriatal pathway from the orbitofrontal cortex (OFC) to the striatum. Before ADSHE onset (four weeks of age), functional abnormalities of glutamatergic transmission compared to the wild-type in the cortical hyperdirect pathway, from OFC to the subthalamic nucleus (STN) in S286L-TG, could not be detected. Conversely, after ADSHE onset (eight weeks of age), glutamatergic transmission in the hyperdirect pathway of S286L-TG was enhanced compared to the wild-type. Notably, enhanced glutamatergic transmission of S286L-TG was revealed by hemichannel activation in the OFC. Expression of connexin43 (Cx43) in the OFC of S286L-TG was upregulated after ADSHE onset but was almost equal to the wild-type prior to ADSHE onset. Differences in the expression of phosphorylated protein kinase B (pAkt) before ADSHE onset between the wild-type and S286L-TG were not observed; however, after ADSHE onset, pAkt was upregulated in S286L-TG. Conversely, the expression of phosphorylated extracellular signal-regulated kinase (pErk) was already upregulated before ADSHE onset compared to the wild-type. Both before and after ADSHE onset, subchronic nicotine administration decreased and did not affect the both expression of Cx43 and pErk of respective wild-type and S286L-TG, whereas the pAkt expression of both the wild-type and S286L-TG was increased by nicotine. Cx43 expression in the plasma membrane of the primary cultured astrocytes of the wild-type was increased by elevation of the extracellular K^+^ level (higher than 10 mM), and the increase in Cx43 expression in the plasma membrane required pErk functions. These observations indicate that a combination of functional abnormalities, GABAergic disinhibition, and upregulated pErk induced by the loss-of-function S286L-mutant α4β2-nAChR contribute to the age-dependent and sleep-induced pathomechanism of ADSHE via the upregulation/hyperactivation of the Cx43 hemichannels.

## 1. Introduction

In 1994 [1], the first gene mutation associated with idiopathic epilepsy was identified in a pedigree of autosomal-dominant sleep-related hypermotor epilepsy (ADSHE) [2]. Over the past two decades, numerous gene mutations associated with various idiopathic epilepsies have been identified [3]. Until recently, the gene mutations associated with ADSHE were also identified in several genes, such as *CHNRA2, CHRNA4, CHRNB2, CRH, KCNT1,* and *DEPDC5* [2,4,5]. In spite of these efforts, neither the detailed pathomechanisms nor the pathophysiologies of ADSHE have been clarified. To explore pathomechanisms of ADSHE, several genetic ADSHE animal models bearing a missense mutation in the *Chrna4* gene, including S280F, insL, and S284L (classical ADSHE-mutations), have been generated. We also studied the pathomechanism/pathophysiology of ADSHE, using transgenic ADSHE rat models, namely S284L-TG, V286L-TG, and S286L-TG [6,7,8,9,10,11]. Functional analysis, using S286L-TG, which bears an S286L-mutation in the rat *Chrna4* gene encoding the α4 subunit of the nicotinic acetylcholine (ACh) receptor (nAChR) corresponding to the S284L-mutation in the human *CHRNA4* gene of ADSHE, demonstrated several important functional abnormalities associated with ADSHE and its comorbidity of cognitive deficit [6,7,8,12]. 

Recently, we demonstrated two candidate fundamental functional abnormalities associated with the epileptogenesis/ictogenesis of S286L-TG. The first was the loss-of-function S286L-mutant α4β2-nAChR (enhanced α4β2-nAChR desensitization and ACh sensitivity), which attenuates GABAergic inhibition in the intrathalamic pathways from the reticular thalamic nucleus (RTN) to motor thalamic nuclei or the mediodorsal thalamic nucleus (MDTN) [6,7,8,12]. The second were the expressions of connexin43 (Cx43) in the frontal cortex (secondary motor cortex) and thalamus (MDTN), which are predominant expression regions of α4-nAChR subunit; they were upregulated in S286L-TG compared with the wild-type littermates [6,12].

The first functional abnormality, impaired GABAergic inhibition in motor thalamic nuclei, plays important roles in the pathomechanisms of typical ADSHE seizures, electroencephalogram (EEG)-sensitive “episodic nocturnal wandering”, and “nocturnal paroxysmal arousal” [7,9] via enhanced glutamatergic transmission in the thalamocortical motor pathway (from motor thalamic nuclei to the secondary motor cortex) [6,8]. Impaired GABAergic inhibition in the motor thalamic nuclei also contributes to the pathomechanisms of EEG-insensitive “nocturnal paroxysmal dystonia” via enhanced glutamatergic transmission in the thalamic hypermotor pathway from the motor thalamic nuclei to the subthalamic nucleus (STN), but not via the cortical hyperdirect pathway (from the secondary motor cortex to the STN) [6,8]. Therefore, “nocturnal paroxysmal dystonia” is generated by abnormal intrathalamic GABAergic transmission similar to other EEG-sensitive ADSHE seizure phenotypes, but this may indicate a paroxysmal movement disorder rather than epileptic seizures, as during nocturnal paroxysmal dystonia, the neuronal hyperexcitability is restricted within the thalamus and basal ganglia and does not propagate to the cortex [6,7,8,12]. These previous studies demonstrated the pathomechanisms of EEG-sensitive “episodic nocturnal wandering” and “nocturnal paroxysmal arousal”, as well as EEG-insensitive “nocturnal-paroxysmal-dystonia”; however, these three ADSHE seizures can be comorbid or complicated in the same episodes among individuals. In particular, clinically, “nocturnal paroxysmal dystonia” occurs as an EEG-insensitive paroxysmal movement disorder independently, but it is not rare for this disorder to precede the development of EEG-sensitive “episodic nocturnal wandering” and “nocturnal paroxysmal arousal” [2,13]. Furthermore, even during “episodic nocturnal wandering”, it is not rare to be unable to detect ictal discharge when the orbitofrontal cortex (OFC) is the origin of the ictus [2,13,14]. ADSHE seizures often feature stereotypical dystonic postures [2,13]. Therefore, the clinical evidence suggests that the epileptic circuits of EEG-insensitive “nocturnal paroxysmal dystonia”, EEG-sensitive “episodic nocturnal wandering”, and “nocturnal paroxysmal arousal” are likely connected with each other through unknown circuits. In other words, these complicated movement features between EEG-sensitive and EEG-insensitive ADSHE phenotypes suggest that the hyperexcitabilities among the thalamus, cortex, and basal ganglia are likely integrated. According to our hypothesis, to better understand the detailed pathomechanism of ADSHE with human S284L-mutations, we should clarify the transmission abnormalities between the cortex and basal ganglia. In the basal ganglia, both the striatum and STN receive the glutamatergic terminal from cortical glutamatergic inputs through the corticostriatal and cortical hyperdirect pathways [15,16]. Our previous study did not detect any connectivity between the secondary motor cortex and STN in wild-type and S286L-TG [8]. Cortical connectivity to the STN is sparser compared to that to the striatum, but OFC exhibits higher tract strength for the STN relative to the striatum [17]. Therefore, based on the established neuronal pathway, to explore the functional abnormalities between the frontal cortex and basal ganglia, the present study determined transmission in the corticostriatal (OFC-striatum) and cortical hyperdirect (OFC-STN) pathways, using multi-probe microdialysis systems with an ultra-high-performance liquid chromatograph (UHPLC). 

The second functional abnormality that Cx43 expresses in the frontal (secondary motor cortex) and thalamic (MDTN) plasma membrane fraction of S286L-TG was shown to be upregulated rather than the wild-type [6,12]. Furthermore, among wild-type, the activation of α4β2-nAChR suppresses Cx43 expression in the plasma membrane fraction of the secondary motor cortex; however, α4β2-nAChR-induced suppression of Cx43 expression could not be observed in S286L-TG [6]. Connexins are the constituent molecules of hemichannel and Gap-junctions [18]. Cx43 is the most widely and predominantly expressed connexin isoform in astrocytes [18,19]. The hemichannel/connexon is assembled with six connexins, and the connexon on the plasma membrane in two neighboring neurons, along with astrocytes, oligodendrocytes, and microglia, forms a gap-junction [18]. The gap-junction plays important roles in physiological functions, including neuronal excitability, synaptic plasticity, tripartite synaptic transmission, and homeostasis maintenance in the central nervous system [18,19]. Astroglial hemichannels also regulate ionic homeostasis, including ionic movement regulation between intracellular and extracellular spaces and the release of several gliotransmitters, including adenosine triphosphate (ATP), nicotinamide adenine dinucleotide, L-glutamate, and prostaglandins, which are involved in autocrine/paracrine signaling [18,20]. During the resting stage, the astroglial hemichannels do not contribute to gliotransmitter release due to their low opening probability [18,20,21]; however, activated hemichannels induced by depolarization and/or extracellular/intracellular cation mobilization (elevations in extracellular K^+^ and reductions in extracellular Ca^2+^) generate gliotransmitter release [18,20,21]. Therefore, hyperactivated astroglial hemichannels are considered to contribute to the generation of epileptic discharges [19,22]. 

Upregulated Cx43 in the thalamus and frontal cortex are activated by repetitive/sustained neuronal excitabilities, such as interictal/ictal discharges or physiological benign discharge (sleep spindles) [6,12,23]. The enhanced glutamatergic input to the secondary motor pathway plays important roles in the generation of ADSHE focus due to the hyperactivation of the upregulated Cx43 containing hemichannel [6,7]. Therefore, the mechanism by which the loss-of-function S286L-mutant α4β2-nAChR enhances Cx43 expression in the plasma membrane is another scientific issue that must be clarified. Binding of the agonist to α4β2-nAChR leads to opening with the desensitization of its cation channel, resulting in rapid depolarization on the order of milliseconds. Besides such electrophysiological rapid responses, nAChRs are also recognized to affect long-term intracellular signaling via several pathways [24,25]. The effects of nAChRs on intracellular signaling have been energetically studied as therapeutic targets for Alzheimer’s disease and carcinoma [24,25,26,27]. It has been established that Cx43 expression in the plasma membrane is regulated by various posttranscriptional processes, including phosphorylation, acetylation, nitrosylation, sumoylation, and ubiquitylation [18,28]. Our previous study, using S284L-TG, demonstrated that, before ADSHE onset, chronic administration of furosemide prevents ADSHE onset via the upregulation of the K^+^/2Cl^-^ cotransporter (KCC) [10]; however, another line of vitro studies also demonstrated that furosemide inhibits mitogen-activated protein kinase (MAPK)/extracellular signal-regulated kinase (Erk) signaling [29]. The Cx43 expression/function is also regulated by several protein kinases, including MAPK/Erk and phosphoinositide 3-kinase (PI3K)/protein kinase B (Akt) pathways [18,30,31,32,33,34]. The activation of α7-nAChR enhances the signaling of PI3K/Akt and MAPK/Erk [35,36], whereas the effects of α4β2-nAChR on this signaling remains to be clarified [25]. Therefore, to explore the mechanisms of upregulation of Cx43 in S286L-TG, the effects of the subchronic administration of nicotine on the expression of Cx43, phosphorylated Akt (pAkt), and phosphorylated Erk (pErk) were also explored, using a capillary immunoblotting system. 

## 2. Results

### 2.1. Glutamatergic Transmission Abnormality in the Hyperdirect and Cirticostriatal Pathways Associated with the Hemichannel before and after ADSHE Onset

It is well-known that, during the resting stage, the hemichannel has low opening probability, but an extracellular cation condition, increased K^+^, and decreased Ca^2+^ levels activates hemichannel activity [6,12,20,21,37]. According to previous demonstrations, to study the activated hemichannel activity on tripartite synaptic transmission in the OFC, the perfusion medium in the OFC was switched from modified Ringers solution (MRS) to Ca^2+^-free with 100 mM K^+^ containing modified Ringer’s solution (FCHK-MRS), for 20 min (FCHK-evoked stimulation) [6,8]. To explore the interaction between the effects of hemichannel and α4β2-nAChR on glutamatergic transmission in the cortical hyperdirect (OFC-STN) and corticostriatal (OFC-striatum) pathways of the wild-type and S286L-TG, before (four weeks of age) and after (eight weeks of age) ADSHE onset, the perfusion medium in the OFC began by using MRS with or without (control) 100 μM carbenoxolone (CBX: hemichannel inhibitor), 100 μM (E)-N-Methyl-4-(3-pyridinyl)-3-buten-1-amine oxalate (RJR2406: selective α4β2-nAChR agonist), or 100 μM CBX plus 100 μM RJR2406. The perfusates in the STN and striatum were maintained with MRS, alone, during the experiment. After the stabilization of the L-glutamate level in the STN or the striatum, the perfusate in the OFC was switched to MRS containing the same agent with 100 μM amino-3-(3-hydroxy-5-methyl-isoxazol-4-yl)propanoic acid (AMPA) for 180 min (first AMPA-evoked stimulation). After the first AMPA-evoked stimulation, the perfusion medium in the OFC was switched to MRS. After the stabilization of the L-glutamate level in the STN or striatum, the perfusion medium in the OFC was switched to FCHK-MRS (Ca^2+^-free with 100 mM K^+^) for 20 min (hemichannel activation). After the stabilization of the L-glutamate level in the STN or striatum, the perfusion medium in the OFC was switched to MRS containing the same agent with 100 μM AMPA, for 180 min again (second AMPA-evoked stimulation). The interval between the first and second AMPA-evoked stimulations was around 240 min. The detailed experimental designs are indicated in Section 4.3.

Notably, CBX (non-selective hemichannel and gap-junction inhibitor) rapidly/reversibly inhibits these channels, and is widely used but offer lower permeability through the plasma membrane [38]. Therefore, CBX is a hemichannel blocker rather than a gap-junction inhibitor; however, 100 μM CBX inhibited the voltage-gated sodium channel [39]. In the present study, CBX was administered, using the reverse dialysis technique [40,41]. The estimated penetration ratio of CBX (molecular weight is 570.8) from the intra to extra dialysis probe was lower than 10% [42]. Therefore, the concentration of CBX in the brain tissue around the dialysis probe was lower than 10 μM. 

#### 2.1.1. Glutamatergic Transmission Abnormality in the Hyperdirect and Cirticostriatal Pathways Associated with the Hemichannel after ADSHE Onset (Eight Weeks of Age) (Study_1)

**Figure 1:**
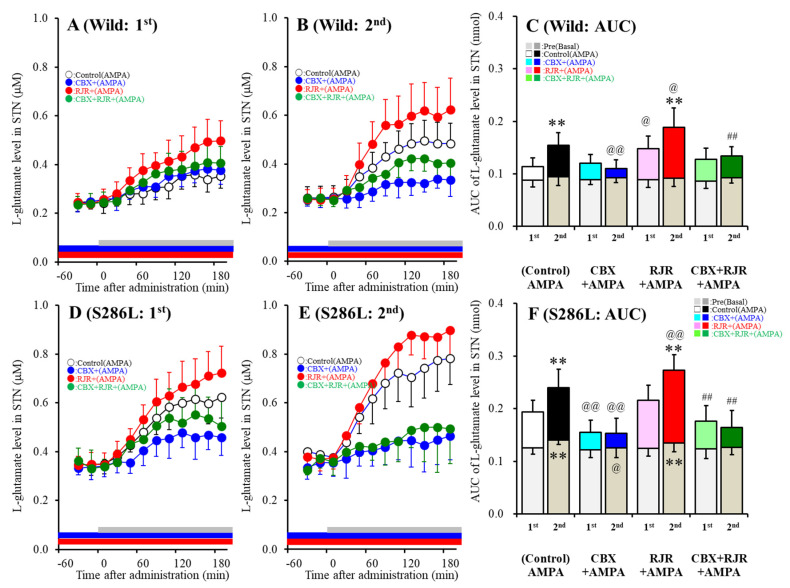
Effects of the local administration of 100 μM RJR2403 (selective α4β2-nAChR agonist) and 100 μM carbenoxolone (CBX; hemichannel inhibitor) into the orbitofrontal cortex (OFC) on 100 μM amino-3-(3-hydroxy-5-methyl-isoxazol-4-yl)propanoic acid (AMPA)-evoked (perfusion with 100 μM AMPA into the OFC) L-glutamate release in the subthalamic nucleus (STN), before (**A**,**D**) and after (**B**,**E**) hemichannel activation (FCHK-MRS (Ca^2+^-free with 100 mM K^+^ containing modified Ringer’s solution) perfusion) of the wild-type (**A**,**B**) and S286L-TG (**D**,**E**). The perfusion medium in the OFC began with MRS, with or without (control) 100 μM CBX (blue bars) or 100 μM RJR2403 (red bars). After stabilization of the L-glutamate level in the STN, the perfusion medium was switched to the same MRS containing 100 μM AMPA, for 180 min (first AMPA-evoked stimulation: gray bars). After stabilization of the L-glutamate level in the STN, the perfusion medium was switched from MRS to FCHK-MRS, for 20 min (hemichannel activation). After stabilization of the L-glutamate level in the STN, the perfusion medium was again switched to the same MRS containing 100 μM AMPA, for 180 min (second AMPA-evoked stimulation: gray bars). Ordinates (**A**,**B**,**D**,**E**) indicate the mean extracellular L-glutamate level (μM) (*n* = 6), and abscissas indicate the time after AMPA-evoked stimulations (min). (**C**,**F**) indicate the area under the curve (AUC) value of the extracellular L-glutamate level (nmol) before (basal extracellular L-glutamate level) and during perfusion with AMPA (from 20 to 180 min) of the wild-type (**A**,**B**) and S286L-TG (**D**,**E**). Notably, the gray columns in (**C**,**F**) indicate the AUC values of the basal extracellular levels of L-glutamate before AMPA-evoked stimulation (basal L-glutamate release) (over −60 to 0 min in (**A**,**B**,**D**,**E**)). * *p* < 0.05, ** *p* < 0.01; relative to the first (first AMPA-evoked stimulation), @*p* < 0.05, @@*p* < 0.01; relative to the control, # *p* < 0.05, ## *p* < 0.01; relative to RJR by MANOVA with Tukey’s multiple comparison. The F-values of the L-glutamate level in the STN, according to a multivariate analysis of variance (MANOVA), were F_event_(1,80) = 15.1(*p* < 0.01), F_RJR_(1,80) = 17.3(*p* < 0.01), F_CBX_(1,80) = 76.8(*p* < 0.01), F_genotype_(1,80) = 128.6 (*p* < 0.01), F_event*RJR_(1,80) = 0.2 (*p* > 0.05), F_event*CBX_(1,80) = 21.3(*p* < 0.01), F_event*genotype_(1,80) = 0.1(*p* > 0.05), F_RJR*CBX_(1,80) = 1.8(*p* > 0.05), F_RJR*genotype_(1,80)= 0.1(*p* > 0.05), F_CBX*genotype_(1,80) = 13.9(*p* < 0.01), F_event*RJR*CBX_(1,80) = 0.1(*p* > 0.05), F_event*RJR*genotype_(1,80) = 0.1 (*p* > 0.05), F_event*CBX*genotype_(1,80) = 0.6(*p* > 0.05), F_RJR*CBX*genotype_(1,80) = 0.1(*p* > 0.05), and F_event*RJR*CBX*genotype_ (1,80)= 0.7 (*p* > 0.05).

The basal extracellular L-glutamate level in the STN of S286L-TG was larger than that of the wild-type (Figure 1). Perfusion with 100 μM AMPA into the OFC increased L-glutamate release in the STN (first AMPA-evoked release) (Figure 1A,D) of S286L-TG, which was larger than that of the wild-type (Figure 1C,F). Hemichannel activation in the OFC induced by perfusion of FCHK-MRS into the OFC for 20 min did not affect the basal L-glutamate level in the STN of the wild-type (Figure 1A–C) but increased that of S286L-TG (Figure 1D–F), which was reduced by perfusion with 100 μM CBX (hemichannel inhibitor) into the OFC (Figure 1D–F). Hemichannel activation in the OFC enhanced the release of 100 μM second AMPA-evoked L-glutamate in the STN of both wild-type and S286L-TG (Figure 1C,F). Inhibition of the hemichannel by perfusion with 100 μM CBX into the OFC inhibited the first AMPA-evoked L-glutamate release in the STN of S286L-TG, without affecting that of the wild-type (Figure 1C,F); however, perfusion with CBX inhibited the second AMPA-evoked L-glutamate release in the STN of both wild-type and S286L-TG (Figure 1C,F). Activation of α4β2-nAChR in the OFC induced by perfusion with 100 μM RJR2406 into the OFC enhanced both the first and second AMPA-evoked L-glutamate release in the STN of the wild-type, without affecting that of the S286L-TG (Figure 1C,F). 

**Figure 2:**
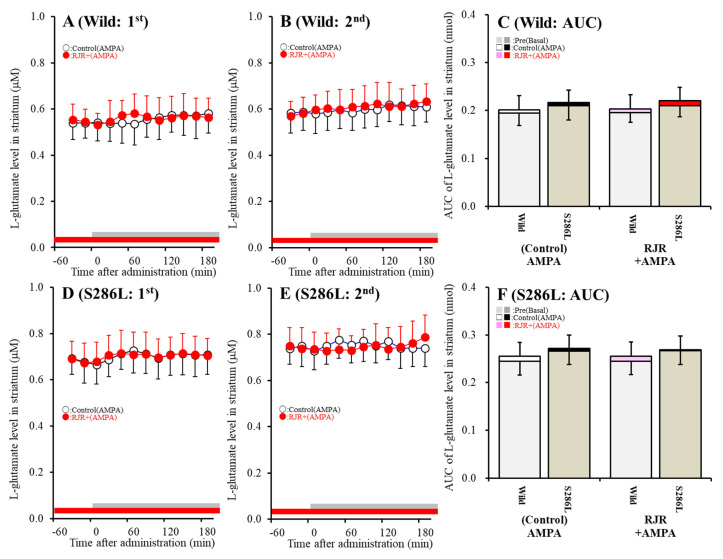
Effects of the local administration of 100 μM RJR2403 into the OFC on 100 μM AMPA-evoked (perfusion with 100 μM AMPA into the OFC) L-glutamate release in the striatum, before (**A**,**D**) and after (**B**,**E**) hemichannel activation (FCHK-MRS perfusion for 20 min) of the wild-type (**A**,**B**) and S286L-TG (**D**,**E**). The perfusion medium in the OFC began with MRS with or without (control) 100 μM RJR2403 (red bars). After stabilization of the L-glutamate level in the striatum, the perfusion medium was switched to the same MRS containing 100 μM AMPA for 180 min (first AMPA-evoked stimulation: gray bars). After stabilization of the L-glutamate level in the striatum, the perfusion medium was switched from MRS to FCHK-MRS for 20 min (hemichannel activation). After stabilization of the L-glutamate level in the striatum, the perfusion medium was again switched to the same MRS containing 100 μM AMPA for 180 min (second AMPA-evoked stimulation: gray bars). Ordinates (**A**,**B**,**D**,**E**) indicate the mean extracellular L-glutamate level (μM) (*n* = 6), and abscissas indicate the time after AMPA-evoked stimulations (min). (**C**,**F**) indicate the AUC of the extracellular L-glutamate level (nmol) before (basal extracellular L-glutamate release) and during perfusion with AMPA (from 20 to 180 min) of the wild-type (**A**,**B**) and S286L-TG (**D**,**E**), respectively. Notably, the gray columns in (**C**,**F**) indicate the AUC values of the basal extracellular levels of L-glutamate before AMPA-evoked stimulation (basal L-glutamate release) (over −60 to 0 min in (**A**,**B**,**D**,**E**)). The F-values of the L-glutamate level in the striatum by MANOVA were F_event_(1,40) = 3.8(*p* > 0.05), F_RJR_(1,40) = 0.1(*p* > 0.05), F_genotype_(1,40) = 40.1(*p* < 0.01), F_event*RJR_(1,40) = 0.1(*p* > 0.05), F_event*genotype_(1,40) = 0.1 (*p* > 0.05), F_RJR*genotype_(1,40) = 0.1 (*p* > 0.05), F_RJR*genotype_(1,40) = 0.1(*p* > 0.05), and F_event*RJR*genotype_ (1,40) = 0.1 (*p* > 0.05).

Contrary to the STN, neither the L-glutamate level in the striatum of S286L-TG nor the wild-type was affected by perfusion with 100 μM AMPA and 100 μM RJR2403 into the OFC; however, the basal L-glutamate level in the striatum of S286L-TG was larger compared to that of the wild-type (Figure 2). Therefore, the present study could not detect a corticostriatal (OFC-striatum) glutamatergic pathway, whereas glutamatergic neurons are present in the OFC project terminals to the STN (cortical hyperdirect pathway). The cortical hyperdirect glutamatergic transmission receives excitatory α4β2-nAChR in the OFC in the wild-type, whereas the S286L-mutant α4β2-nAChR in the OFC cannot affect cortical hyperdirect glutamatergic transmission. However, the cortical hyperdirect pathway in S286L-TG was regulated by the activated hemichannel in the OFC compared to that of the wild-type. 

#### 2.1.2. Glutamatergic Transmission Abnormality in the Hyperdirect and Cirticostriatal Pathways Associated with the Hemichannel before ADSHE Onset (Four Weeks of Age) (Study_2)

**Figure 3:**
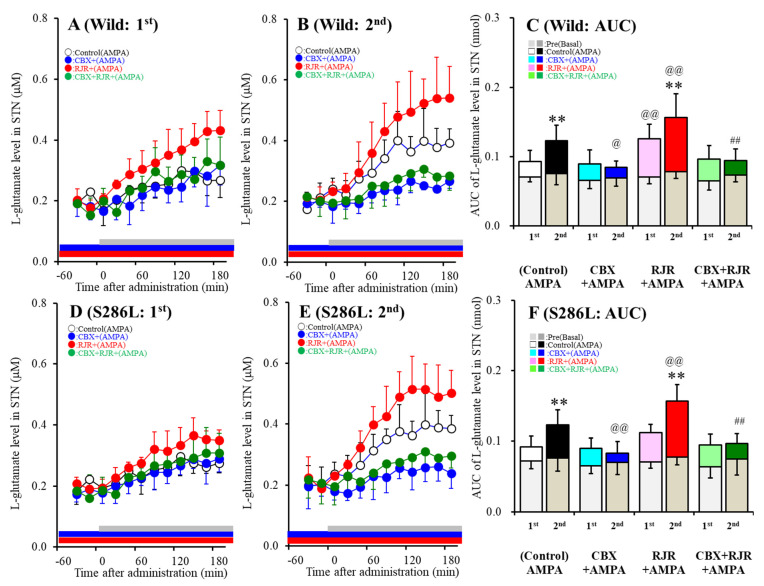
Effects of the local administration of 100 μM RJR2403 (selective α4β2-nAChR agonist) and 100 μM CBX (hemichannel inhibitor) into the OFC on 100 μM AMPA-evoked (perfusion with 100 μM AMPA into the OFC) L-glutamate release in the STN, before (**A**,**D**) and after (**B**,**E**) hemichannel activation (FCHK-MRS (Ca^2+^-free with 100 mM K^+^) perfusion) of the wild-type (**A**,**B**) and S286L-TG (**D**,**E**). Ordinates (**A**,**B**,**D**,**E**) indicate the mean extracellular L-glutamate level (μM) (*n*=6), and abscissas indicate the time after AMPA-evoked stimulations (min). (**C**,**F**) indicate the AUC value of the extracellular L-glutamate level (nmol) before (basal extracellular L-glutamate level) and during perfusion with AMPA (from 20 to 180 min) for the wild-type (**A**,**B**) and S286L-TG (**D**,**E**), respectively. Especially, gray columns in (**C**,**F**) indicate the AUC values of the basal extracellular levels of L-glutamate before AMPA-evoked stimulation (basal L-glutamate release) (during −60 to 0 min in **A**,**B**,**D**,**E**). * *p* < 0.05, ** *p* < 0.01; relative to the first (first AMPA-evoked stimulation), @*p* < 0.05, @@*p* < 0.01; relative to the control, # *p* < 0.05, ## *p* < 0.01; relative to RJR by MANOVA with Tukey’s multiple comparison. The F-values of the L-glutamate level in the STN, according to a multivariate analysis of variance (MANOVA), were F_event_(1,80) = 15.1 (*p* < 0.01), F_RJR_(1,80) = 21.9 (*p* < 0.01), F_CBX_(1,80) = 59.9 (*p* < 0.01), F_genotype_(1,80) = 0.2 (*p* > 0.05), F_event*RJR_(1,80) = 0.7 (*p* > 0.05), F_event*CBX_(1,80) = 19.3 (*p* < 0.01), F_event*genotype_(1,80) = 0.3 (*p* > 0.05), F_RJR*CBX_(1,80) = 6.6 (*p* < 0.05), F_RJR*genotype_(1,80) = 0.1 (*p* > 0.05), F_CBX*genotype_(1,80) = 0.1 (*p* > 0.05), F_event*RJR*CBX_(1,80) = 0.1 (*p* > 0.05), F_event*RJR*genotype_(1,80) = 0.4 (*p* > 0.05), F_event*CBX*genotype_(1,80) = 0.1 (*p* > 0.05), F_RJR*CBX*genotype_(1,80) = 0.2 (*p* > 0.05), and F_event*RJR*CBX*genotype_(1,80)= 0.1 (*p* > 0.05).

Contrary to after ADSHE onset (eight weeks of age), the basal extracellular L-glutamate level in the STN of S286L-TG prior to ADSHE seizure onset (four weeks of age) was almost equal to that of the wild-type (Figure 3). Both the first and second AMPA-evoked stimulation into the OFC increased L-glutamate release in the STN (Figure 3A,D). The first AMPA-evoked L-glutamate release in the STN of S286L-TG and the wild-type were also almost equal (Figure 3C,F). Perfusion with 100 μM CBX into the OFC did not affect the first AMPA-evoked L-glutamate release but decreased the second AMPA-evoked L-glutamate release in the STN of both S286L-TG and the wild-type (Figure 3C,F). Perfusion with 100 μM RJR2406 increased the first AMPA-evoked L-glutamate release in the STN of the wild-type but did not affect that of the S286L-TG; however, the second AMPA-evoked L-glutamate released in the STN of both S286L-TG and the wild-type were enhanced by 100 μM RJR2406 (Figure 3C,F). Perfusion with 100 μM CBX suppressed the stimulatory effects of 100 μM RJR2406 on the second AMPA-evoked L-glutamate release in the STN of both genotypes (Figure 3C,F). Therefore, before ADSHE onset, there were some functional abnormalities in L-glutamate release associated with the hemichannels, whereas the stimulatory effects of α4β2-nAChR on AMPA-evoked L-glutamate release were impaired in S286L-TG. 

#### 2.1.3. Tetrodotoxin (TTX) and CBX-Sensitive Basal L-Glutamate Release in the OFC of S286L-TG after Hemichannel Activation (Study_3)

**Figure 4:**
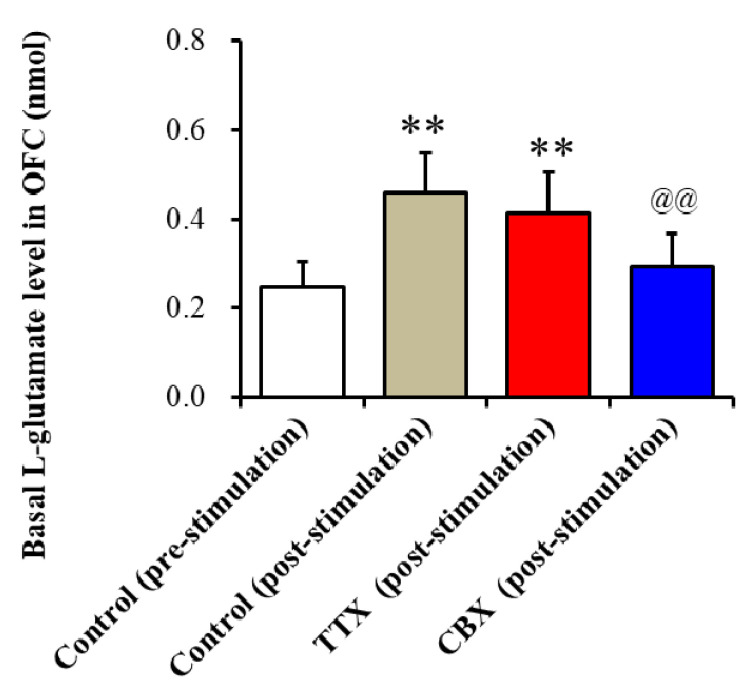
Effects of the local administration of 1 μM tetrodotoxin (TTX) and 100 μM CBX into the OFC on basal L-glutamate release in the OFC of S286L-TG after FCHK-evoked stimulation. The perfusion medium in the OFC was switched to MRS, with or without (control: post-stimulation: gray column) 1 μM TTX (red column) or 100 μM CBX (blue column). The ordinate AUC of the extracellular L-glutamate level (nmol) for 60 min during perfusion of MRS with or without TTX or CBX is shown. ** *p* < 0.01, relative to the control (pre-stimulation), and @@ *p* < 0.01, relative to the control (post-stimulation) by a one-way analysis of variance (ANOVA) with Tukey’s multiple comparison. The F-values of the L-glutamate level in the OFC, according to the one-way ANOVA, are F(3,20) = 9.6 (*p* < 0.01).

To clarify the mechanisms of enhanced glutamatergic transmission in the cortical hyperdirect pathway induced by hemichannel activation in the OFC of ADSHE onset S286L-TG after FCHK-evoked stimulation in the OFC (after the Study_1), the perfusion medium in the OFC was switched to MRS containing 1 μM TTX (voltage-dependent sodium channel inhibitor) [43] or 100 μM CBX (hemichannel inhibitor). The increased basal L-glutamate release in the OFC after FCHK-evoked stimulation was decreased by CBX but not affected by TTX (Figure 4). Therefore, the increased basal L-glutamate release in the STN of S286L-TG induced by FCHK-evoked stimulation was considered to be of astroglial hemichannel origin but not of neuronal exocytosis origin. 

### 2.2. Effects of Subchronic Nicotine Administration on Cx43 Expression in the OFC 

**Figure 5:**
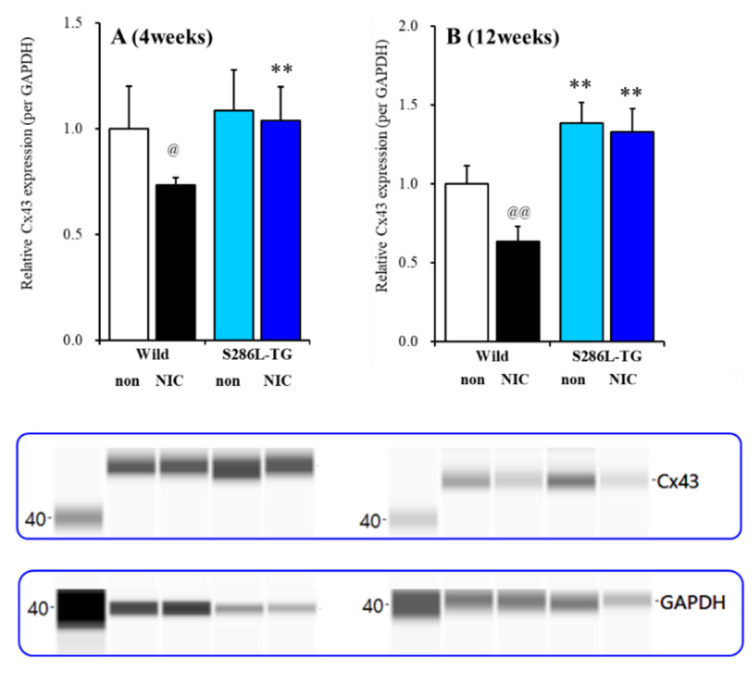
Effects of subchronic nicotine administration on connexin43 (Cx43) expression in the OFC. Effects of systemic subchronic administration of nicotine (50 mg/kg/day for seven days) on Cx43 expression in the OFC plasma membrane fraction before four weeks of age (**A**), and after 12 weeks of age (**B**), autosomal dominant sleep-related hypermotor epilepsy (ADSHE) onset of the wild-type and S286L-TG and pseudo-gel images from the capillary immunoblotting results, using anti-GAPDH and anti-connexin43 antibodies for blotting of the plasma membrane fractions. Ordinate: mean ± SD (*n* = 6) of the relative protein level of Cx43. ** *p* < 0.01 vs. the wild-type, and @ *p* < 0.05, @@ *p* < 0.01 vs. nicotine-free (non) based on a two-way ANOVA with Tukey’s multiple comparison.

To study the effects of subchronic administration of nicotine on Cx43 expression in the plasma membrane, S286L-TG and the wild-type rats (3 and 11 weeks of ages) were subchronically administrated nicotine (50 mg/kg/day for seven days), using a subcutaneous osmotic pump (2ML_1, Alzet), according to a previous study [6,12,23]. At four weeks of age (before ADSHE onset), Cx43 expression in the OFC plasma membrane fraction of S286L-TG and the wild-type was almost equal, whereas the subchronic administration of nicotine (50 mg/kg/day for seven days) decreased the Cx43 expression of the wild-type but did not affect that of S286L-TG (F_genotype_(1,20) = 8.8 (*p* < 0.01), F_nicotine_(1,20) = 5.7 (*p* < 0.05), or F_genotype* nicotine_(1,20) = 2.7 (*p* > 0.05)) (Figure 5A). Unlike prior to ADSHE onset, at 12 weeks of age (after ADSHE onset), the Cx43 expression of S286L-TG was larger than that of the wild-type (Figure 5B). Subchronic administration of nicotine (50 mg/kg/day for seven days) decreased Cx43 expression of the wild-type but did not affect that of S286L-TG (F_genotype_(1,20) = 118.0 (*p* < 0.01), F_nicotine_(1,20) = 17.8 (*p* < 0.01), and F_genotype*nicotine_(1,20) = 9.6 (*p* < 0.01)) (Figure 5B).

### 2.3. Effects of Subchronic Nicotine Administration on Akt and Erk Expression in the OFC 

**Figure 6:**
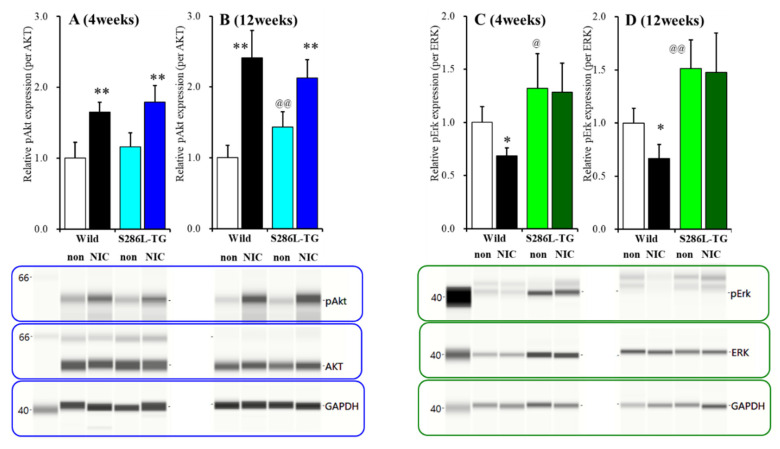
Effects of subchronic nicotine administration on the expression of phosphorylated protein kinase B (pAkt) and phosphorylated extracellular signal-regulated kinase (pErk) in the plasma membrane fraction of OFC. Effects of the systemic subchronic administration of nicotine (50 mg/kg/day for seven days) on pAkt and pErk expression in the OFC plasma membrane fraction before four week of age (**A**,**C**) and after 12 week of age (**B**,**D**), ADSHE onset of the wild-type and S286L-TG and pseudo-gel images, using capillary immunoblotting. Ordinate: mean ± SD (*n* = 6) of the relative protein level of pErk and pAkt. * *p* < 0.05, ** *p* < 0.01 vs. wild-type, and @ *p* < 0.05, @@ *p* < 0.01 vs. nicotine-free (non) by two-way ANOVA with Tukey’s multiple comparison.

At four weeks of age (before ADSHE onset), phosphorylated Akt (pAkt) expression in the OFC plasma membrane fraction of S286L-TG was almost equal to that of the wild-type; however, at 12 weeks of age (after ADSHE onset), pAkt expression in the OFC plasma membrane fraction of S286L-TG was larger than that of the wild-type (Figure 6A,B). Subchronic administration of nicotine (50 mg/kg/day for seven days) increased pAkt expression of the wild-type and S286L-TG before (F_genotype_(1,20) = 3.5 (*p* > 0.05), F_nicotine_(1,20) = 61.3 (*p* < 0.01), and F_genotype*nicotine_(1,20) = 0.1 (*p* > 0.05)) and after (F_genotype_(1,20) = 0.1 (*p* > 0.05), F_nicotine_(1,20) = 103.3 (*p* < 0.01), and F_genotype*nicotine_(1,20) = 18.2 (*p* < 0.01)) the ADSHE onset periods (Figure 6A,B).

At four weeks of age, phosphorylated Erk (pErk) expression in the OFC plasma membrane fraction of S286L-TG was already larger than that of the wild-type, whereas subchronic administration of nicotine (50 mg/kg/day for seven days) decreased pErk expression of the wild-type but did not affect that of S286L-TG (F_genotype_(1,20) = 24.6 (*p* < 0.01), F_nicotine_(1,20) = 3.7 (*p* > 0.05), and F_genotype*nicotine_(1,20) = 2.3 (*p* > 0.05)) (Figure 6C). Like before ADSHE onset, at 12 weeks of age S286L-TG was larger than that of the wild-type (Figure 6D). Subchronic administration of nicotine (50 mg/kg/day for seven days) decreased pErk expression of the wild-type but did not affect that of S286L-TG (F_genotype_(1,20) = 42.3 (*p* < 0.01), F_nicotine_(1,20) = 3.3 (*p* > 0.05), and F_genotype*nicotine_(1,20) = 2.1 (*p* > 0.05)) (Figure 6D).

### 2.4. Effect of Erk and Extracellular K^+^ Level on Astroglial Cx43 Expression 

After culturing for 28 days (DIV28) to study the effects of the extracellular K^+^ level on Cx43 expression in the plasma membrane, the cultured medium was changed from a Dulbecco’s modified Eagle’s medium containing 10% fetal calf serum (fDMEM) to N-fDMEM (control: fDMEM plus 4.6 mM NaCl: 5.4 mM K^+^), MK-fDMEM (fDMEM plus 2.1 mM KCl and 2.5 mM NaCl: 7.5 mM K^+^), and HK-fDMEM (fDMEM plus 4.6 mM KCl: 10.0 mM K^+^) for 6 h (around the half-life of Cx43 [44]). To study the effects of Akt and Erk on Cx43 expression in the plasma membrane, the cultured medium was changed to HK-fDMEM containing 20 μM FR180204 (Erk inhibitor) or 10 μM 10-DEBC (Akt inhibitor) for 6 h. 

**Figure 7:**
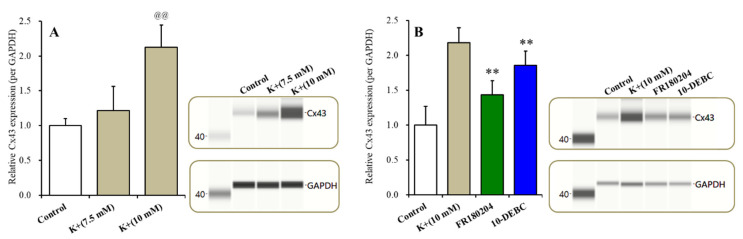
Effects of subacute administration of an increase in the extracellular K^+^ level on Cx43 expression in the plasma membrane fraction of primary cultured astrocytes (**A**). Effects of the inhibitor of Erk (FR180204) and Akt (10-DEBC) on K^+^-dependent Cx43 expression in the plasma membrane (**B**). Ordinate: mean ± SD (*n* = 6) of the relative protein level of Cx43. Concentration-dependent effects of extracellular K^+^ on Cx43 expression in the plasma membrane fraction of the primary cultured astrocytes were analyzed by a one-way ANOVA (@@ *p* < 0.01 vs. control). The effects of 20 μM FR180204 and 10 μM 10-DEBC on Cx43 expression in the plasma membrane fraction were analyzed by a Student’s *t*-test (** *p* < 0.01 vs. 10 mM K^+^).

Cx43 expression in the plasma membrane fraction of the wild-type primary cultured astrocytes increased the extracellular K^+^ level concentration-dependently (F(2,15) =27.2 (*p* < 0.01)) (Figure 7A). MK-fDMEM (7.5 mM K^+^) did not affect Cx43 expression, whereas HK-fDMEM (10.0 mM K^+^) increased Cx43 expression in the plasma membrane fraction (Figure 6A). The concentration-dependent expression of the extracellular K^+^ of Cx43 in the plasma membrane was suppressed by the inhibitor of both Erk (FR180204) and Akt (10-DEBC) (*p* < 0.01) (Figure 7B).

## 3. Discussion

### 3.1. Mechanisms of Upregulation of Cx43 of S286-TG.

We have already demonstrated that a combination of attenuated intrathalamic GABAergic transmission and upregulated/hyperactivated Cx43 in the secondary motor cortex and thalamus of S286L-TG plays important roles in the pathomechanisms of ADSHE [6,7,8,12]. The propagation of epileptic discharge in the thalamocortical cognitive (MDTN-OFC) pathway was highly dependent upon Cx43 upregulation/hyperactivation in the thalamus compared to that in the thalamocortical motor pathway (from the motor thalamic nuclei to the secondary motor cortex) [8,12]. The present study also detected the upregulation of Cx43 in the OFC plasma membrane of S286L-TG. 

Activation of α4β2-nAChR suppressed Cx43 expression [6,45]; however, the loss-of-function S286L-mutant α4β2-nAChR could not affect Cx43 expression in S286L-TG [6,12]. Trafficking of Cx43 to the plasma membrane was regulated by various posttranscriptional processes (phosphorylation, acetylation, nitrosylation, sumoylation, and ubiquitylation) [18,28], including PI3K/Akt and MAPK/Erk signaling [18,34,46]. Indeed, in the present study, the inhibitors of MAPK/Erk (FR180204) and PI3K/Akt (10-DEBC) suppressed Cx43 expression in the plasma membrane fraction of the primary cultured astrocytes [33,46]. Homomeric α7-nAChR stimulated proliferation via the activation of the signaling of PI3K/Akt and MAPK/Erk [24,25,35,36]. Contrary to α7-nAChR, α4β2-nAChR inhibits proliferation, but its effects on PI3K/Akt and MAPK/Erk signaling remain to be clarified [24,25,26]. Nicotine inhibits the mRNA of MAPK and PI3K, but the inhibitory effects of nicotine administration were attenuated age-dependently [27]. These age-dependent effects of nicotine on intracellular signaling associated with the PI3K/Akt and MAPK/Erk pathways suggest possible involvement in the age-dependent pathomechanisms of ADSHE. Based on these previous findings, we hypothesized that Cx43 upregulation in α4β2-nAChR-predominant expression regions, the thalamus and frontal cortex of S286L-TG, is likely generated by abnormalities in signaling associated with PI3K/Akt and/or MAPK/Erk induced by the loss-of-function S286L-mutant α4β2-nAChR.

According to our expectations, before ADSHE onset (four weeks of age), Cx43 expression in the plasma membrane of S286L-TG OFC was almost equal to that of the wild-type; however, after ADSHE onset (12 weeks of age), Cx43 expression in the plasma membrane of S286L-TG was upregulated compared to that of the wild-type. Subchronic nicotine administration reduced Cx43 expression of the wild-type at both 4 and 12 weeks of age, whereas nicotine did not affect the Cx43 expression of S286L-TG before and after ADSHE onset. These results suggest that the loss-of-function S286L-mutant α4β2-nAChR plays important roles in the pathomechanisms of ADSHE via the upregulation of astroglial Cx43. 

Contrary to Cx43 expression, the pErk of S286L-TG was already upregulated before ADSHE onset compared to that of the wild-type. Subchronic nicotine administration reduced the pErk of the wild-type at both 4 and 12 weeks of age, whereas nicotine did not affect the pErk of S286L-TG before and after ADSHE onset. Furthermore, our previous study already demonstrated that furosemide, which prevents the ADSHE onset of S284L-TG [10], inhibits MAPK/Erk signaling [29]. Thus, considering our previous demonstration, the upregulation of pErk preceding Cx43 upregulation in the OFC of S286L-TG suggests the possible pathomechanisms of ADSHE though which the upregulation of MAPK/Erk signaling induced by the loss-of-function S286L-mutant α4β2-nAChR plays key roles in the development of the epileptogenesis of ADSHE.

Differences between pAkt expression of the wild-type and S286L-TG were not observed before the ADSHE onset periods; however, after ADSHE onset, the pAkt of S286L-TG was upregulated compared to that of the wild-type. Conversely, sub-chronic nicotine administration increased the pAkt of both wild-type and S286L-TG before and after the ADSHE onset periods. This nicotine-induced upregulation of pAkt is generated by the nicotine-induced activation of α7-nAChR, which is consistent with a previous demonstration [24,25,35,36]. Whether the upregulation of pAkt after ADSHE onset is pathomechanism or a result of ADSHE seizure is a fundamental neuroscientific issue. It is well-known that enhanced IP3K/Akt signaling plays a key role in the development of the epileptogenesis and ictogenesis of various epileptic syndromes via suppression of the tuberous sclerosis complex (TSC) and activation of mammalian target of rapamycin (mTOR) signaling [47]. In other line aspects, epileptic seizure also upregulates PI3K/Akt/mTOR signaling [48]. Based on these previous findings, the present study cannot assert that pAkt is not involved in the pathomechanism of ADSHE, but the upregulation of pErk may play a more important role in the development of the epileptogenesis of ADSHE than in pAkt signaling.

### 3.2. Impact of the Upregulation/Activation of Cx43 in ADSHE.

During the resting stage, the astroglial hemichannel exhibits a low opening probability [6,12,20,21] but is activated by the depolarization of membrane potential and specific fluctuations in the extracellular and intracellular cation levels [6,12,21]. A functional analysis study using S284L-TG demonstrated that impaired GABAergic inhibition and interictal discharge onset are observable at four and six weeks of age (both before ADSHE onset), respectively [10,11]. Electrophysiological studies of Xenopus oocytes, using voltage-clamps, showed that the S284L-mutant α4β2-nAChR enhances ACh-sensitivity and desensitization [49]. Therefore, the enhanced irritability (repetitive/persistent excitability) induced by impaired GABAergic inhibition via the loss-of-function S286L-mutant α4β2-nAChR can activate hemichannel functions [6,12]. In our previous microdialysis studies, 25 mM K^+^-evoked stimulation that could increase neurotransmitter release [50], but gliotransmitter release required greater than 100 mM extracellular K^+^ levels [12,37]. However, elevation of the extracellular K^+^ level around 10–12 mM plays an important role in the generation of hypersynchronous neuronal excitability, and epileptic discharge also increases the extracellular K^+^ level to over 10 mM [51]. Based on these previous findings, to explore the repetitive/persistent elevation of the extracellular K^+^ level (over 10 mM) on astroglial hemichannel activity, the effects of subacute (for 6 h, longer than the half-life of Cx43 [44]) exposure to the 10 mM extracellular K^+^ level on Cx43 expression in the plasma membrane of the wild-type primary cultured astrocytes were studied. According to our expectations, astroglial Cx43 expression was increased by elevation of the extracellular K^+^ level (7.5 mM K^+^ did not affect Cx43 expression, but the threshold level of 10 mM K^+^ increased). Taken together with our previous findings, the present demonstrations suggest multiple age-dependent and event-induced (sleep and epileptic seizure) stages of pathomechanisms in ADSHE. 

Therefore, the congenital loss-of-function S286L-mutant α4β2-nAChR generates two functional abnormalities in S286L-TG, GABAergic disinhibition [10,11] and upregulation of the MAPK/Erk signaling pathway. Impaired GABAergic inhibition leads to the relative enhancement of glutamatergic transmission in the thalamocortical and hyperdirect pathways in α4β2-nAChR predominant regions [6,7,8,12], resulting in the generation of interictal discharge before ADSHE onset [11]. A combination of hyperglutamatergic transmission (repetitive/persistent propagation of discharges) and an upregulated MAPK/Erk signaling pathway contributes to the development of epileptogenesis/ictogenesis via the upregulation of astroglial Cx43.

It is well-known that the majority of ADSHEs (50–60%) can be controlled by a relatively low dose of carbamazepine, whereas ADSHE with an S284L mutation has a carbamazepine-resistant feature and requires other antiepileptic drugs, such as zonisamide [4,41,52,53]. A therapeutically relevant concentration of zonisamide inhibits both the activity and expression of Cx43 in the astroglial plasma membrane; however, a therapeutically relevant concentration of carbamazepine does not affect these factors [23,54]. Therefore, the activated hemichannel in the OFC likely also contributes to the pathophysiology/ictogenesis of carbamazepine-resistant/zonisamide-sensitive ADSHE seizures with S284L-mutations. 

### 3.3. Neural Circuits Associated with Dystonia Posturing in ADSHE Seizures

In our previous study, hyperactivated glutamatergic transmission in the thalamic hyperdirect pathway (from motor thalamic nuclei to the STN), but not the glutamatergic transmission abnormalities in the cortical hyperdirect pathway (from the secondary motor cortex to the STN), provided the pathomechanism of nocturnal paroxysmal dystonia [6]. The cortical connectivity to the STN is sparser compared to that to the striatum, but the OFC exhibits higher tract strength for the STN relative to the striatum [17]. A clinical study observed OFC seizures during nocturnal paroxysmal dystonia and episodic nocturnal wandering featuring stereotypical dystonic posturing [13]. In another ADSHE model, S280F-knockin mice strain also exhibited a dystonic/arousal complex, which is a complex between the nocturnal paroxysmal dystonia and nocturnal paroxysmal arousal of ADSHE seizures [55]. These clinical and preclinical findings suggest that hyperactivity in the basal ganglia is possibly involved in ADSHE seizures. Therefore, to explore the pathomechanisms of ADSHE seizures, the present study determined glutamatergic transmission abnormalities in the cortical hyperdirect (OFC-STN) and corticostriatal (OFC-striatum) pathways. 

According to our expectations, after ADSHE onset (eight weeks of age), activation of the postsynaptic AMPA/glutamate receptor in the OFC increased L-glutamate release in the STN (cortical hyperdirect pathway) without affecting that in the striatum (corticostriatal pathway) in both wild-type and S286L-TG. Hemichannel activation in the OFC enhanced L-glutamate release in the STN of both wild-type and S286L-TG, but sensitivity to the hemichannel activation of S286L-TG was predominant rather than that of the wild-type. Interestingly, the glutamatergic transmission in the cortical hyperdirect pathway associated with the astroglial hemichannel was observed prior to hemichannel activation in S286L-TG, since CBX inhibited AMPA-evoked L-glutamate release before FCHK-evoked stimulation. Taken together with the upregulation of Cx43 expression in the OFC of S286L-TG, at eight weeks of age, the upregulated Cx43 hemichannel of S286L-TG was probably weakly activated during the interictal stages. Contrary to the hemichannel, in the wild-type, α4β2-nAChR enhanced AMPA-evoked glutamate release in the cortical hyperdirect pathway under conditions of both hemichannel resting and activation in the OFC, whereas the AMPA-evoked L-glutamate release in the STN of S286L-TG was insensitive to α4β2-nAChR before hemichannel activation. These discrepancies in responses to the α4β2-nAChR of glutamatergic transmission in the hyperdirect pathway between S286L-TG and the wild-type suggest that the loss-of-function S286L-mutant α4β2-nAChR likely does not directly contribute to the generation of epileptic ADSHE focus in the OFC, but indirectly contributes to the generation of focus via the activation/upregulation of Cx43 hemichannel hyperactivation.

Similar to the observations at eight weeks of age, the activation of α4β2-nAChR enhanced AMPA-evoked glutamate release in the cortical hyperdirect pathway under conditions of both hemichannel resting and activation in the OFC of the wild-type, whereas before hemichannel activation, the AMPA-evoked L-glutamate release of S286L-TG was insensitive to α4β2-nAChR. In contrast to α4β2-nAChR, the sensitivity levels to the activated hemichannel of glutamatergic transmission in the cortical hyperdirect pathway of S286L-TG showed similar features to the wild-type. Therefore, the differences in the sensitivity of glutamatergic transmission to activated hemichannels before (four weeks of age) and after (eight weeks of age) ADSHE seizure onset suggest that the activation of upregulated Cx43 in the OFC of S286L-TG likely contributes to the development of ictogenesis through the hyperactivation of tripartite synaptic transmission. In other words, the hypersensitivity of hemichannel activity in the OFC to the propagation of discharges (physiological sleep spindle, interictal and ictal discharges) plays important roles in clinical ADSHE features, such as the common occurrence of ADSHE seizures during non-REM sleep phases and once ADSHE seizure leading to subsequent frequent seizures during the same night [2,13].

## 4. Materials and Methods 

### 4.1. Chemical Agents and Drug Administration

The (E)-N-Methyl-4-(3-pyridinyl)-3-buten-1-amine oxalate (RJR2403: selective α4β2-nAChR agonist) was obtained from Cosmo Bio (Tokyo, Japan). The Amino-3-(3-hydroxy-5-methyl-isoxazol-4-yl)propanoic acid (AMPA: AMPA/glutamate receptor agonist), voltage-dependent sodium channel inhibitor, tetrodotoxin (TTX), and nicotine ditartrate were obtained from Wako Chemicals (Osaka, Japan). Carbenoxolone (CBX: hemichannel inhibitor) and 10-[4’-(N,N-diethylamino)butyl]-2-chlorophenoxazine hydrochloride (10-DEBC: Akt inhibitor) were obtained from Funakoshi (Tokyo, Japan). Moreover, 5-(2-Phenyl-pyrazolo [1,5-a]pyridin-3-yl)-1H-pyrazolo[3,4-c]pyridazin-3-ylamine (FR180204: Erk inhibitor) was obtained from Tokyo Chemical Industry (Tokyo, Japan).

All compounds were prepared on the day of the experiment. In the microdialysis study, AMPA, CBX, TTX, and RJR2403 were dissolved in a modified ringer solution (MRS) composed of the following (in mM): 145 Na^+^, 2.7 K^+^, 1.2 Ca^2+^, 1.0 Mg^2+^, and 154.4 Cl^−^, buffered with 2 mM phosphate buffer and 1.1 mM Tris buffer at pH 7.4 [56,57,58,59,60]. In the primary cultured astrocyte study, FR180204 was initially made as 10 mM stocks in dimethyl sulfoxide and then diluted to Dulbecco’s modified Eagle’s medium (D6546: Sigma-Aldrich, St. Louis, MO) containing 10% fetal calf serum (fDMEM). The 10-DEBC was dissolved in fDMEM directly. To study the effects of subchronic nicotine administration on the expression of Cx43, pErk/Erk, and pAkt/Akt in the OFC plasma membrane, rats were subchronically administered with nicotine ditartrate (50 mg/kg/day for 7 days), using a subcutaneous osmotic pump (2ML_1, Alzet, Cupertino, CA; the nominal pumping rate and duration were 10 μL/h over 7 days).

### 4.2. Experimental Animals

Animal care, experimental procedures, and protocols for the animal experiments were approved by the Animal Research Ethics Committee of the Mie University School of Medicine (No. 24-37-R3, 7 March 2018). All studies involving animals were reported in accordance with the ARRIVE guidelines for reporting experiments involving animals. A total 126 rats, wild-type littermates (*n* = 90), and S286L-TG rats (*n* = 84) [7,61] (Sprague Dawley strain background, SLC, Shizuoka, Japan) were maintained in a controlled environment (22 ± 1 °C) on a 12 h dark/light cycle and used in the experiments as described. Rats were randomly assigned to the treatment groups of each experiment. All experiments in this study were designed with equally sized animal groups (*n* = 6) without carrying out a formal power analysis, in keeping with previous studies [6,7,8,12]. Where possible, we sought to randomize and blind the data. In particular, for the determination of L-glutamate and protein levels, the sample order was determined by a random number table.

### 4.3. Microdialysis

Wild-type (*n* = 60) and S286L-TG (*n* = 60) rats (4 and 8 weeks of age) were anesthetized with 1.8% isoflurane and then placed on a stereotactic frame. Microdialysis studies were prepared, using a protocol adapted from previously described methods [6,7,8,12,54,58,62]. A concentric direct-insertion type dialysis probe (0.22 mm diameter, 3 mm exposed membrane: Eicom, Kyoto, Japan) was implanted in the OFC (A = +3.2 mm, L = +2.4 mm, V = −6.5 mm, relative to bregma) and striatum (A = +1.0 mm, L = −3.5 mm, V = −6.0 mm, relative to bregma) [7,62,63,64]. Another concentric direct-insertion type probe with a shorter exposed membrane (0.22 mm diameter, 1 mm exposed membrane: Eicom) was then implanted in the STN (A = −3.6 mm, L = −2.5 mm, V = −8.4 mm, relative to bregma) [8,63,65,66]. Experiments were not started until three consecutive baseline transmitter measurements yielded a coefficient of variation of less than 5%. Dialysates were then collected for 60 min (pretreatment period) followed by 180 min of sampling after AMPA administration [6,12].

For the microdialysis study, to activate the hemichannel function in the OFC, the perfusion medium in the OFC was switched to Ca^2+^-free with 100 mM K^+^ containing MRS (FCHK-MRS) for 20 min (FCHK-MRS activation) [6,8] (Figure 1). To explore the effects of the hemichannel and α4β2-nAChR on glutamatergic transmission in the cortical hyperdirect (OFC-STN) and corticostriatal (OFC-striatum) pathways of the wild-type and S286L-TG, the perfusion medium in the OFC began with MRS with or without (control) 100 μM CBX (non-selective hemichannel inhibitor), 100 μM RJR2406 (selective α4β2-nAChR agonist), or 100 μM CBX plus 100 μM RJR2406. The perfusates in the STN and striatum were maintained with MRS alone during the experiment. After stabilization of the L-glutamate level in the STN or striatum, the perfusate in the OFC was switched to MRS containing the same agent with 100 μM AMPA for 180 min (1st AMPA-evoked stimulation). After the 1st AMPA-evoked stimulation, the perfusion medium in the OFC was switched to MRS. After stabilization of the L-glutamate level in the STN or striatum, the perfusion medium in the OFC was switched to FCHK-MRS (Ca^2+^-free with 100 mM K^+^) for 20 min (hemichannel activation). After stabilization of the L-glutamate level in the STN or striatum, the perfusion medium in the OFC was switched to MRS containing the same agent with 100 μM AMPA for 180 min again (2nd AMPA-evoked stimulation) (Figure 1). The time between the 1st and 2nd AMPA-evoked stimulations was around 240 min.

### 4.4. Ultra-High-Performance Liquid Chromatography (UHPLC)

L-glutamate levels were determined by using UHPLC equipped with xLC3185PU (Jasco, Tokyo, Japan) and fluorescence detection (xLC3120FP, Jasco) following dual derivatization with isobutyryl-L-cysteine/o-phthalaldehyde [8,67,68]. The derivatized samples (5 μL aliquots) were injected via an autosampler (xLC3059AS, Jasco). The analytical column (YMC Triat C18, particle 1.8 μm, 50 × 2.1 mm, YMC, Kyoto, Japan) was maintained at 45 °C, and the flow rate was set to 500 μL/min. A linear gradient elution program was used over a period of 10 min with mobile phases A (0.05 M citrate buffer, pH 5.0) and B (0.05 M citrate buffer containing 30% acetonitrile and 30% methanol, pH 3.5). The excitation/emission wavelengths of the fluorescence detector were set to 280/455 nm.

### 4.5. Capillary Immunoblotting Analysis

The capillary immunoblotting analysis was performed, using Wes (ProteinSimple, Santa Clara, CA, USA), according to the ProteinSimple user manual [6,7,8,12]. The lysates of the primary cultured astrocytes were mixed with a master mix (ProteinSimple) to a final concentration of 1 × sample buffer, 1 × fluorescent molecular weight marker, and 40 mM dithiothreitol and then heated at 95 °C for 5 min. The samples, blocking reagents, primary antibodies, HRP-conjugated secondary antibodies, chemiluminescent substrate (SuperSignal West Femto: Thermo Fisher Scientific, Waltham, MA, USA), and separation and stacking matrices were also dispensed to the designated wells in a 25 well plate. After plate loading, the separation electrophoresis and immunodetection steps took place in the capillary system and were fully automated. A capillary immunoblotting analysis was carried out at room temperature, and the instrument’s default settings were used. Capillaries were first filled with a separation matrix followed by a stacking matrix, with about 40 nL of the sample used for loading. During electrophoresis, the proteins were separated by molecular weight through the stacking and separation matrices at 250 volts for 40–50 min and then immobilized on the capillary wall, using proprietary photo-activated capture chemistry. The matrices were then washed out. The capillaries were next incubated with a blocking reagent for 15 min, and the target proteins were immunoprobed with primary antibodies followed by HRP-conjugated secondary antibodies (Anti-Rabbit IgG HRP, A00098, 10 μg/mL, GenScript, Piscataway, NJ). The antibodies of GAPDH (NB300-322, 1:100, Novus Biologicals, Littleton, CO, USA), Cx43 (C6219, 1:100, Sigma-Aldrich, St. Louis, MO, USA), Erk (AF1576, 10 μg/mL, R&D systems, Minneapolis, MN, USA), pErk (AF1018, 5 μg/mL, R&D systems), Akt (AF1775, 1 μg/mL, R&D systems), and pAkt (AF877, 5 μg/mL, R&D systems) were diluted in an antibody diluent (ProteinSimple).

To study the effects of α4β2-nAChR on the expression of Cx43 and Erk in the OFC plasma membrane, both wild-type (*n* = 6) and S286L-TG (*n* = 6) at 4 weeks of age (before ADSHE onset) and 12 weeks of age (after ADSHE onset) [7,11] were subchronically administrated nicotine (50 mg/kg/day for 7 days), using a subcutaneous osmotic pump (2ML_1, Alzet) [6,56,57]. The total plasma membrane proteins of the OFC and primary cultured astrocytes were extracted, using a Minute Plasma Membrane Protein Isolation Kit (Invent Biotechnologies, Plymouth, MN) [6].

### 4.6. Primary Cultured Astrocytes

Astrocytes were prepared, using a protocol adapted from previously described methods [22,54,68]. Pregnant Sprague-Dawley rats (SLC, Sizuoka, Japan) were housed individually in cages and kept in air-conditioned rooms (temperature, 22 ± 2 °C), with a 12 h light/dark cycle and free access to food and water. Cultured astrocytes were prepared from the cortical astrocyte cultures of neonatal Sprague-Dawley rats (*n* = 6) sacrificed by decapitation at 0–24 h of age. Cerebral hemispheres were removed under a dissecting microscope. Tissues were chopped into fine pieces, using scissors and then triturated briefly with a micropipette. The suspension was filtered, using a 70 µm nylon mesh (BD, Franklin Lakes, NJ, USA), and centrifuged. Pellets were then re-suspended in fDMEM, which was repeated three times. After culturing for 14 days (DIV14), the contaminating cells were removed via shaking in a standard incubator (BNA-111, Espec, Osaka, Japan) for 16 h at 200 rpm. On DIV21, the astrocytes were removed from the flasks by trypsinization and seeded directly onto a translucent PET membrane (1.0 μm) with 24 well plates (BD) at a density of 1 × 105 cells/cm^2^ for the experiments. From DIV21 to DIV27, the culture medium (fDMEM) was changed twice a week for 7 days. On DIV27, to study the effects of the extracellular K^+^ level on Cx43 expression in the plasma membrane, the cultured medium was changed (for 6 h) to N-fDMEM (control: fDMEM plus 4.6 mM NaCl: 5.4 mM K^+^), MK-fDMEM (fDMEM plus 2.1 mM KCl and 2.5 mM NaCl: 7.5 mM K^+^), and HK-fDMEM (fDMEM plus 4.6 mM KCl = 10.0 mM K^+^). The composition of NaCl and KCl in fDMEM was modified to maintain isotonicity and ionic strength. To study the effects of Erk and Akt on Cx43 expression in the plasma membrane, the medium was changed (for 6 h) to N-fDMEM containing 20 μM FR180204 (Erk inhibitor) or 10 μM 10-DEBC (Akt inhibitor) for 6 h. On DIV 28, the cultured astrocytes were washed out, using artificial-CSF, and then the total plasma membrane proteins were extracted, using a Minute Plasma Membrane Protein Isolation Kit (Invent Biotechnologies) [6].

### 4.7. Data Analysis

All experiments in this study were designed with equally sized animal groups (*n* = 6), without carrying out a formal power analysis, in keeping with previous studies. All values are expressed as the mean ± SD, and *p* < 0.05 (two-tailed) was considered statistically significant for all tests. Drug levels in acute local and subchronically systemic administrations were selected based on values in previous studies. Where possible, we sought to randomize and blind the data. In particular, for the determination of transmitter levels and protein expression, the sample order on the autosampler and Wes were determined by a random number table.

The regional transmitter concentrations in the STN and striatum were analyzed via a Mauchly’s sphericity test followed by a multivariate analysis of variance (MANOVA), using BellCurve for Excel ver. 3.2 (Social Survey Research Information Co., Ltd., Tokyo, Japan). When the data did not violate the assumption of sphericity (*p* > 0.05), the F-value of the MANOVA was analyzed, using sphericity-assumed degrees of freedom. However, if the assumption of sphericity was violated (*p* < 0.05), the F-value was analyzed, using Chi-Muller’s corrected degrees of freedom. When the F-value for the genotype/drug/time factors of MANOVA was significant, the data were analyzed by a Tukey’s multiple comparison test. The transmitter level was expressed as the area under the curve between 20 and 180 min (AUC_20–180_) after the perfusion of AMPA containing MRS. The effects of perfusion with TTX and CBX into the basal L-glutamate release were analyzed, using a one-way analysis of variance (ANOVA) with Tukey’s multiple comparison. The protein expression of Cx43, pErk, and pAkt in the plasma membrane fraction was analyzed by a two-way ANOVA with Tukey’s multiple comparison, using BellCurve for Excel.

## 5. Conclusions

**Figure 8:**
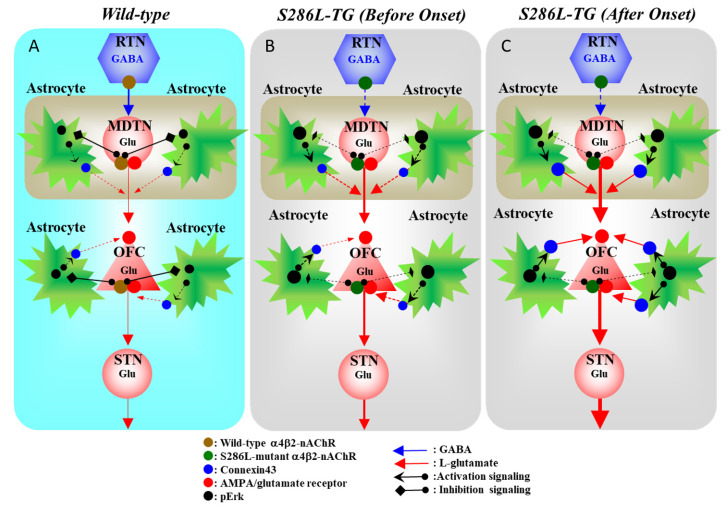
Proposed hypothesis of the multi-stage pathomechanisms of S286L-TG. Proposed hypothesis for the functional abnormalities of glutamatergic transmission in the thalamocortical and cortical hyperdirect pathways in the wild-type (**A**), S286L-TG before ADSHE onset (**B**), and after ADSHE onset (**C**). Reticular thalamic nucleus (RTN) mainly projects GABAergic terminals to various thalamic nuclei, including mediodorsal thalamic nucleus (MDTN). The activation of α4β2-nAChR in the RTN enhances GABAergic transmission in the RTN–MDTN pathways of the wild-type (**A**), whereas the S286L-mutant α4β2-nAChR impairs the activation of GABAergic transmission in the RTN–MDTN in S286L-TG (**B**,**C**). MDTN project glutamatergic terminals to the OFC. In the MDTN, both α4β2-nAChR and the AMPA/glutamate receptor activate glutamatergic transmission to the OFC (**A**–**C**). Wild-type α4β2-nAChR inhibits astroglial Erk, resulting in the suppression of connexin43 expression in the astroglial plasma membrane (**A**). Contrary to the wild-type, in S286L-TG, the loss-of-function S286L-mutant α4β2-nAChR lacks suppressive effects on pErk (**B**,**C**) but is insufficient to upregulate connexin43 (**B**). A combination of the persistent/repetitive propagation of the hyperactivation of glutamatergic transmission in MDTN-OFC induced by the GABAergic disinhibition of S286L-TG and pErk upregulation enhances connexin43 expression.

In conclusion, this study provided evidence for the age-dependent and sleep/seizure-induced multi-stage pathomechanisms of ADSHE with S284L-mutations, using the genetic ADSHE model (S286L-TG). Congenital functional abnormalities and the loss-of-function S286L-mutant α4β2-nAChR produce GABAergic disinhibition, resulting in enhanced glutamatergic transmission and an upregulated MAPK/Erk signaling pathway (Figure 8). Wild-type α4β2-nAChR suppresses pErk, but the S286L-mutant α4β2-nAChR impairs the inhibitory function of pErk, resulting in the upregulation of Cx43 expression. Under functional abnormalities, the propagation of physiological (sleep spindle bursts) and pathological (interictal/ictal discharges) discharges to the OFC leads to an event-related enhancement in the function of upregulated Cx43, resulting in enhanced excitatory tripartite synaptic transmission (Figure 8). Therefore, a combination of secondary functional abnormalities induced by the loss-of-function S286L-mutant α4β2-nAChR, GABAergic disinhibition, and Cx43 upregulation contributes to the pathomechanism of ADSHE. Interestingly, even if ADSHE seizures are controlled, once a patient experiences an ADSHE seizure, he or she will experience many more ADSHE seizures during that same night.

## Abbreviations

10-DEBC	10-[4’-(N,N-Diethylamino)butyl]-2-chlorophenoxazine hydrochloride
Ach	acetylcholine
ADSHE	autosomal dominant sleep-related hypermotor epilepsy
Akt	protein kinase B
AMPA	amino-3-(3-hydroxy-5-methyl-isoxazol-4-yl)propanoic acid
ANOVA	analysis of variance
AUC	area under curve value
CBX	carbenoxolone
CHRNA2	cholinergic receptor nicotinic alpha 2 subunit
CHRNA4	cholinergic receptor nicotinic alpha 4 subunit
CHRNB2	cholinergic receptor nicotinic beta 2 subunit
CRH	corticotropin releasing hormone
Cx43	connexin43
DEPDC5	DEP domain containing 5, GATOR1 subcomplex subunit
DIV	after culture days
EEG	electroencephalogram
Erk	extracellular signal-regulated kinase
FCHK-MRS	Ca^2+^-free with 100 mM K^+^ containing modified Ringer’s solution
fDMEM	Dulbecco’s modified Eagle’s medium containing 10% fetal calf serum
FR180204	5-(2-Phenyl-pyrazolo[1,5-a]pyridin-3-yl)-1H-pyrazolo[3,4-c]pyridazin-3-ylamine
HK-fDMEM	Dulbecco’s modified Eagle’s medium containing 10% fetal calf serum plus 4.6 mM KCl
KCC	K^+^/2Cl^-^ cotransporter
KCNT1	potassium sodium-activated channel subfamily T member 1
MANOVA	multivariate analysis of variance
MAPK	mitogen-activated protein kinase
MDTN	mediodorsal thalamic nucleus
MK-fDMEM	Dulbecco’s modified Eagle’s medium containing 10% fetal calf serum plus 2.1 mM KCl and 2.5 mM NaCl
MRS	modified Ringer’s solution
mTOR	mammalian target of rapamycin
nAChR	nicotinic acetylcholine receptor
N-fDMEM	Dulbecco’s modified Eagle’s medium containing 10% fetal calf serum plus 4.6 mM NaCl
OFC	orbitofrontal cortex
pAkt	phosphorylated protein kinase B
pErk	phosphorylated extracellular signal-regulated kinase
PI3K	phosphoinositide 3-kinase
RJR2403	(E)-N-Methyl-4-(3-pyridinyl)-3-buten-1-amine oxalate
RTN	reticular thalamic nucleus
S286L-TG	bearing S286L-mutation in rat *Chrna4* gene transgenic rat
STN	subthalamic nucleus
TSC	tuberous sclerosis complex
UHPLC	ultra-high-performance liquid chromatograph
